# COVID‐19 transmission through asymptomatic carriers is a challenge to containment

**DOI:** 10.1111/irv.12743

**Published:** 2020-04-15

**Authors:** Xingxia Yu, Rongrong Yang

**Affiliations:** ^1^ Department of Emergency The People's Hospital Wuhan University Wuhan China; ^2^ Department of Infectious Diseases Zhongnan Hospital Wuhan University Wuhan China

To the Editor,

Since the first report on the outbreak of a novel coronavirus disease COVID‐19 in Wuhan, Hubei, China, in December, 2019,^1^ there have been 78 064 cases have been confirmed and 2715 deaths as of February 25, 2020. For any infectious disease, there are three kinds of way to control the epidemic of infectious disease‐that is, to control the source of infection, to cut off transmission routes, and to protect the susceptible population. As a new infectious disease, it is difficult to develop a safe and effective vaccine against COVID‐19 in a short period of time. So, it is not possible to protect susceptible population at present. Social distancing is one of the main ways to cut off transmission routes – people cannot pass on infection if they do not come into contact with other people. Based on the understanding that COVID‐19 spreads through respiratory droplets, there has been widespread use of face masks in Wuhan. The propaganda "no masks, no going outside" has been adopted by the public. This can help to cut off the transmission routes of COVID‐19. Increases in capacity for laboratory testing, allowing treatment and isolation of COVID‐19 patients also helps to reduce onwards transmission. In the period of initial success for COVID‐19 control and prevention, one bottleneck has been screening out asymptomatic carriers for reducing new infections. As frontline doctors, we believe that there have been asymptomatic carriers of COVID‐19 who can infect their close contacts.

Although asymptomatic patients with SARS were uncommon, they were documented in a retrospective study in the minor 2004 SARS outbreak after reopening of the wildlife market in Guangzhou.^2^ Similarly, during the epidemic of COVID‐19 in Wuhan, one asymptomatic child (aged 10 years) who had a history of travel to Wuhan from Shenzhen was described in the report about a family cluster of COVID‐19.^3^ He had radiological ground‐glass lung opacities, and he was RT‐PCR positive for genes encoding the internal RNA‐dependent RNA polymerase and surface spike protein of this novel coronavirus, which were confirmed by Sanger sequencing. The child was virologically confirmed to have an asymptomatic infection. In addition, another family of three who travelled from Wuhan to Guangzhou was reported to be infected with COVID‐19.^4^ The father had clinical symptoms, a decreased lymphocyte count, abnormal chest CT images, and a positive result on qRT‐PCR. It's interesting that the mother and the son were both asymptomatic, with normal lymphocyte counts and chest CT images but positive qRT‐PCR for COVID‐19 infection.

In both cases, asymptomatic carriers were diagnosed by screening after other family members developed symptoms. We presume there would be many other asymptomatic carriers who were never tested. Even if those carriers were in the hospital, perhaps for another reason, would the doctor think of doing a viral nucleic acid test for COVID‐19 in time? If it is not possible to screen these asymptomatic carriers from truly healthy populations, they will be a source of COVID‐19 infection back into the community, which will also pose a huge challenge for COVID‐19 prevention and control.

On February 25, the case of COVID‐19 transmission occurring in a prison in Rencheng district, Jining city, Shandong province has sounded an alarm for us. On February 9, a man was released from prison and returned home. But he was informed to isolate himself because a prison officer was confirmed to have COVID‐19 infection on February 14. In order to avoid isolation and observation, the man absconded from his home. Later, it was confirmed that this man was an asymptomatic carrier, and his brother who had close contact with him became infected with COVID‐19. This case is an example of asymptomatic transmission. One study has reported that the viral load that was detected in the asymptomatic patients was similar to that in the symptomatic patients, which also theoretically suggests the potential transmission of asymptomatic patients.^5^

In Conclusion, we considered that asymptomatic COVID‐19 infection is possible, and person‐to‐person transmission can occur from asymptomatic COVID‐19 carriers to the community. Therefore, vigilant control measures are warranted at this stage of the COVID‐19 epidemic to avoid a resurgence in cases.

## CONFLICT OF INTEREST

We declare no competing interests.

## Author Contribution

**Xingxia Yu:** Conceptualization (equal); Data curation (equal); Formal analysis (equal); Investigation (equal); Methodology (equal); Project administration (equal); Resources (equal); Software (equal); Supervision (equal); Validation (equal); Visualization (equal); Writing‐original draft (equal); Writing‐review & editing (equal). **Rongrong Yang:** Conceptualization (equal); Data curation (equal); Formal analysis (equal); Investigation (equal); Methodology (equal); Project administration (equal); Resources (equal); Software (equal); Supervision (equal); Validation (equal); Visualization (equal); Writing‐original draft (equal); Writing‐review & editing (equal).
